# Supplementary material for: Characterization of Genetically Modified Microorganisms Using Short- and Long-Read Whole-Genome Sequencing Reveals Contaminations of Related Origin in Multiple Commercial Food Enzyme Products
Source: Foods. 2021 Oct 30;10(11):2637. doi: 10.3390/foods10112637 (PMC8624754; doi:10.3390/foods10112637)
Supplement: Supplementary file 1 [file foods-10-02637-s001.zip › foods-1433822-supplementary.pdf]

Supplementary text: Detailed explanation concerning elucidation of genetic make-up of GMM

1. Long-read and hybrid assemblies provide conflicting characterization of the GMM genetic make-up

Unicycler has been reported in literature to be one of the most performant hybrid assembly tools available, delivering highly accurate and complete assemblies (Chen *et al.*, 2020; de Maio *et al.*, 2019). Kurittu *et al.* (2021) described assembly using Unicycler of complete AMR carrying plasmids in *Escherichia coli* and *Klebsiella pneumonia* strains isolated from a variety of food products, while complete genome assemblies were reported for a Shiga Toxin-producing *E. coli* strain originating from salad greens (Fiedler *et al.*, 2020), and for a *Clostridium botulinum* strain isolated from carrot juice (Harris *et al.*, 2021). Berbers *et al.* (2020) demonstrated its potential for WGS analysis of GM bacteria by presenting a complete assembly for a riboflavin overproducing GM *B. subtilis* strain harboring several recombinant plasmids, including one free plasmid and several others integrated into the chromosome, the latter constituting a repetitive region of at least 53 kb.

For isolates Pilsner1-1, Pilsner1-2, and Pilsner2-2, Unicycler ran without issues, but it failed to run to completion on the remaining 7 isolates. Additional analysis (results not shown) indicated that removing all reads that align to the transgenic sequence (i.e. pUB110 sequence) prior to assembly, resolved this issue, allowing Unicycler to run to completion in all cases. This indicates that the problem of Unicycler failing to complete could likely be attributed to the GM nature of the samples. Alternatively, Canu (long-reads only) and hybridSPAdes were therefore used to generate genome assemblies, and could deliver assemblies in all cases.

Table S1 shows the main statistics for all generated assemblies. Figure 1 depicts graphs of the Unicycler, Canu, and hybridSPAdes assemblies of sample Pilsner1-2, which was chosen as representative isolate because it presented the least fragmented assembly. Assembly graphs for the other isolates were similar for each assembly tool, and are shown in Figure S 4. Depending on the used assembler, different putative answers became apparent to the question how many copies of the transgenic construct were present in the genome of the GMM, and whether they were located on a plasmid and/or integrated into the chromosome.

1.1. Unicycler assemblies indicate the transgenic construct is located on a recombinant pUB110 plasmid

For the three cases where Unicycler ran to completion, three consistent genome assemblies were obtained comprising three separate components: a chromosome made up of 11 to 35 contigs, a circular contig of 6,756 bp, and a linear contig of appr. 13.9 kb in length (Figure 1A, Table S 1, Figure S 4). The most complete assembly was obtained for Pilsner1-2, consisting of 13 contigs in total, and containing 98.7% of the assembly within a single contig representing the chromosome. This assembly was consequently used as representative reference for subsequent analysis.

Explorative BLAST analysis of these assemblies indicated that, of all *B. velezensis* genomes publicly available, the genome of the isolates was most similar to that of *B. velezensis* 10075, a strain isolated from food lobster sauce in China. The *B. velezensis* 10075 RefSeq genome is reported as complete, with a GC% of 46.09, and consists of a chromosome (Genbank: CP025939.1) with length 4,326,822 bp, and a second extrachromosomal element annotated as 'plasmid, unnamed' of 13,003 bp (Genbank: CP025940.1). Web-based blastn against the *B. velezensis* genomes in the NCBI RefSeq Genome Database, using the first 1 million bp of the largest contig of the Pilsner1-2 assembly as query, yielded the *B. velezensis* 10075 chromosome as top hit (based on maximum total score), being 100% identical over a query coverage of 95%. This strain was therefore also included in the comparative genomic analysis (see 3.3).

Web-based blastn analysis against the NCBI nucleotide database further showed that the 6,756 bp contig was a recombinant plasmid derived from the pUB110 shuttle plasmid (Figure 2). The pUB110-matching fraction of the plasmid showed nearly 100% sequence identity with nt 7–4,374 of pUB110 (Genbank: M19465.1), which total length is 4,545 bp. The alignment of the native plasmid with the recombinant plasmid is visualized in Figure S 5. The missing part of pUB110 was replaced by a fragment of 2,386 bp in length that was, according to blastn, 100% identical both to nt 1,869,061–1,871,446 of the *B. velezensis* 10075 chromosome, as well as to the according chromosomal region of Pilsner1-2. This region covered a complete coding sequence annotated as a peptidase M4 family protein (also named protease), as well as part of an upstream pseudogene and downstream gene encoding a GNAT family N-acetyltransferase. In the Unicycler assembly graphs, the relative read-depth of this plasmid compared to the largest (chromosomal) contig varied between 197 and 268, in line with the high-copy nature of the plasmid.

Nt 180–13,182 of the linear contig of the Unicycler assembly of pilsner1-2 were 100% identical to the complete 'plasmid unnamed' of *B. velezensis* 10075. Its relative read depth in the Unicycler assembly

graphs ranged from 2.42 to 3.44, hinting at a low-copy presence. However, sequence analysis with PHASTER revealed that this element is probably not a typical plasmid, but more likely a natural plasmidic prophage (Table S8). Although temperate phages in the lysogenic phase are usually integrated into the chromosome of their host, in some cases they can also be found as independently replicating linear or circular plasmid-like elements. A well-studied group of such temperate phages are tectiviruses that specifically target members of the *B. cereus* group. They are 14–15 kbp in length, and their prophages exist and replicate as linear plasmids inside the host cell (Gillis & Mahillon, 2014). Multiple sequence alignment of the linear 13.9 kb contig of isolate pilsner1-2 and a number of these tectiviral *Bacillus* phage sequences revealed significant sequence similarity, suggesting that the 13.9 kbp element was most likely a linear plasmidic phage (Figure S 6). To the best of our knowledge, association of a tectivirus, in particular a plasmidic prophage, with a member of the *B. subtilis* group to which *B. velezensis* belongs, has not been reported previously.

The Unicycler assemblies of samples Pilsner1-1, and Pilsner2-2 were very similar to that of Pilsner1-2, and also contained the recombinant pUB110-derived plasmid, with exactly the same length of 6,756 bp, as well as the putative plasmidic prophage.

1.2. HybridSPAdes assemblies indicate that the recombinant plasmid is integrated into the chromosome

HybridSPAdes succeeded in providing a hybrid assembly for all 10 isolates, which were however much more fragmented than the Unicycler assemblies, being made up of 165–195 contigs (Figure 1B, Figure S 4, Table S 1). In all but one (i.e. of the cob9-1 isolate) of the hybridSPAdes assemblies, a separate linear contig of 14,052–14,060 bp was found matching the putative plasmidic prophage of *B. velezensis* 10075 identified in the Unicycler assemblies. However, the recombinant pUB110 plasmid was not present in any of the assemblies. Instead, the pUB110 sequence was part of the chromosomal scaffold, present on one or several (depending on the assembly) separate contigs, and associated with the chromosomal wild-type copy of the protease. The hybridSPAdes assemblies hence placed the transgenic pUB110-protease construct on the chromosome, which was not observed for any of the Unicycler assemblies, but did not allow establishing how many copies of the construct were present.

1.3. Canu assemblies indicate the presence of concatemers of the recombinant pUB110 plasmid

Canu long-read assemblies were created successfully for all isolates (Figure 1C, Table S 1, Figure S 4). The assemblies consisted of 4–15 contigs, and their total size ranged between 4.53–4.86 Mbp, which is markedly larger than that of the Unicycler or hybridSPAdes assemblies. It was also substantially larger than any of the publicly available *B. velezensis* chromosomes, which vary in length from 3.71–4.32 Mbp, indicating a certain degree of misassembly by Canu. In four out of ten assemblies, the putative plasmidic prophage was present as a separate contig of 14,040–14,055 bp. Its absence from the six other assemblies can potentially be attributed to the apparent low-copy nature of this element. Strikingly, all assemblies contained one or more contigs that were entirely made up of head-to-tail copies, i.e. concatemers, of the recombinant pUB110-derived plasmid described in section S1.1 with a copy number between 3–11 (Figure 1D). The aforementioned recombinant plasmid itself - in monomer form - was however not detected in any of the Canu assemblies, neither as a free plasmid, nor integrated into the chromosome.

Several other long-read and hybrid assemblers were explored as well, but the results were in all cases inferior to the Canu and/or Unicycler assemblies, and none of them could deliver a qualitative assembly allowing to establish which of the above options represented the ground truth for the genomic organization of the isolates (results not shown).

1.4. AMR genes are associated with the transgenic construct

Resistance characterization using genotypic AMR detection with the assemblies detected two AMR genes; *aadD*, conferring kanamycin and neomycin resistance, and *ble*, conferring bleomycin resistance. In the three Unicycler assemblies, these genes were located on the recombinant plasmid (Figure 2), while in the Canu assemblies the AMR genes were detected on the plasmid concatemer contigs. In the hybridSPAdes assemblies, all detected AMR genes were exclusively associated with the transgenic construct, which in this case was present within the chromosomal scaffold, as described in section S1.2. No AMR genes were detected on the putative plasmidic prophage. Exploratory resistance characterization of a few other wildtype *B. velezensis* genomes, e.g. 10075 and CBMB205 (Genbank: NZ_CP011937) (data not shown), confirmed that absence of AMR genes appears to be common to this species, which would be an asset considering its potential for use in food applications.

2. Additional in-depth BLAST-and-filtering analysis of raw long read data allows to genetically characterize the GMM

2.1. Confirmation of the presence of a free high-copy recombinant plasmid

Since genome assembly did not allow unambiguously characterizing the GMM isolates with respect to the location and copy number of the transgenic construct, long-read data was subjected to additional analyses in an attempt to elucidate these conflicting results.

First, raw long-read data was filtered to retain only reads matching the pUB110 plasmid sequence. Remarkably, read-length histograms of the filtered data showed, for all isolates, a distinct peak at 6.6–6.8 kbp (Figure S 8), corresponding to the length of the recombinant plasmid of 6,756 bp presented in the Unicycler assemblies, hence providing strong support for the presence of the transgenic construct on a free high-copy number plasmid. Additional experimental confirmation of the presence of this plasmid was provided by Illumina sequencing and short-read assembly of a plasmid extract of isolate Cob9-1 (Figure S7). The assembly contained several low read-depth contigs that can be attributed to the presence of residual genomic DNA of *B. velezensis* in the extract. According to web-based blastn against the NCBI nucleotide database, the largest contig, with a length of 60,332 bp, matched most closely to the *B. velezensis* 10075 chromosome, showing 97.23% sequence identity over a query coverage of 84%. The recombinant pUB110 derived plasmid of 6,756 bp was present in the assembly at a read depth of 1,944-fold relative to the read depth of the largest contig, and its sequence was a full-length 100% identical match to that of the recombinant plasmid from the WGS Unicycler assembly of sample Pilsner1-2, depicted in Figure 2.

2.2. Chromosomal integration of the recombinant plasmid is unstable

Subsequently, a BLAST search with the same query was performed, but this time only on reads longer than 6,756 bp, which yielded between 141 to 4,645 pUB110-matching reads per sample. The BLAST hits were visualized with Kablammo (Figure S 9), which demonstrated that the large majority was entirely made up of recombinant plasmid concatemers, similar to the contigs that turned up in Canu assemblies, with between 2 to 12 copies in the raw reads present.

If these concatemers would represent true multi-copy number integrations of the plasmid into the chromosome, we would expect to also find ample reads that cover the junctions between the chromosomal sequence of the isolates and the transgenic construct. However, these were hardly

detected. In a limited number of reads, a putative junction site encompassing chromosomal and transgenic DNA was observed, indicating the integration of the plasmid at the site of the wild-type chromosomal protease encoding gene. Sporadically, raw reads were found for which upstream and/or downstream regions of the transgenic construct appeared to point to integration at other chromosomal sites, but in none of those cases, the same chromosomal region was found more than once, pointing to a chimeric origin of the raw reads in question.

Next, BLAST and filtering were combined in a nested approach to retain only reads covering both the pUB110 sequence and chromosomal sequence flanking the wild-type protease gene. The resulting dataset was expected to contain all reads that covered a junction of the chromosomal integration of the recombinant plasmid. These reads were thereafter mapped to the longest read retrieved as described in the previous paragraph (representing a two-copy integration of the plasmid), with 100% of the reads being mapped for all samples. Figure 3 shows the resulting alignment, which illustrates two observations. Firstly, on average, only 6–7 raw reads were found for each isolate that covered the putative junction. This was one to two orders of magnitude lower than what would be expected based on overall coverage. Since one of the critically-acclaimed advantages of ONT sequencing is its robust sequencing efficiency, regardless of GC content (Browne *et al.*, 2020; Krishnakumar *et al.*, 2018), this drastic coverage difference is unlikely to be due solely to sequencing bias related to the lower GC%-content of the inserted transgenic construct. Nonetheless, enough reads were found over all isolates combined to reject these reads being chimeric constructs. Secondly, detailed analysis of the mapping result showed that the reads supported at least two types of integrations, namely a one-copy integration of the plasmid, which was supported by the majority of the reads, as well as a two-copy head-to-tail integration of the plasmid. Figure 3 shows that only in three cases (marked with blue arrows), a mapped read unambiguously confirmed the presence of a 2-copy plasmid integration. In most cases however, aligned reads did support a pUB110 integration event, but not necessarily the two-copy insertion, because they were either too short to span two copies of pUB110, or were soft-clipped at the start of a second pUB110 copy.

Overall, these observations strongly suggest that the transgenic construct was primarily carried on a free high-copy plasmid. Unstable transient chromosomal integration did appear to occur to some limited extent, which is not entirely unexpected given the homologous regions between the plasmid and chromosome.

To verify this hypothesis experimentally, we performed qPCR on genomic DNA of GM isolate Cob9-1, targeting the junction of this chromosomal integration, which yielded a negative signal, notwithstanding

a relatively high amount of genomic DNA (10 ng) was used as template. Despite this negative result, the coverage found in the long read datasets for the integration site, albeit low, was significant, and is best explained, in our opinion, by an unstable integration event that was only transiently present and/or in a small fraction of the cells, rather than by a very unlikely collection of chimeric reads or any other artefact that would occur by chance. In line with our observations, it was previously reported that chromosomal integrations of pUB110 derived plasmids can be unstable, if the plasmid itself replicates stably in the strain, which is generally the case for *Bacillus* strains (Leenhouts *et al.*, 1990). Compared to the native pUB110-plasmid, only a fragment of the *mob* gene was lacking from the recombinant plasmid (Figure 2), whereas all elements required for normal replication (Viret & Alonso, 1988) had remained intact, indicating that the vector was designed to be episomal rather than integrative (Deckers *et al.*, 2020).

2.3. Plasmid concatemers are likely a consequence of disturbed plasmid replication

This leaves the issue of the plasmid concatemers, which were, as mentioned above, abundantly present in the raw long read data of all isolates. However, this phenomenon is not new, and has been extensively described and studied previously. Plasmids that replicate through a rolling-circle replication mechanism, such as pUB110, are known to produce linear plasmid concatemers under certain conditions, such as phage infection, certain genetic host backgrounds, or insertion of foreign sequences (Gruss & Ehrlich, 1988; Viret *et al.*, 1991). Gruss and Ehrlich (1988) reported that insertion of foreign DNA into pUB110 can trigger accumulation of high-molecular-weight head-to-tail plasmid multimers in e.g. *B. subtilis*. Their description of these events is in agreement with our own observations. Additionally, we did not find evidence for junctions that would indicate that these concatemers are integrated into the chromosome (with the exception of the sporadic one- to two-copy integration described above). This phenomenon consequently indicated that the observed plasmid concatemer long reads could be explained by disrupted plasmid replication in cells. This phenomenon, which was likely caused by the GM nature of the isolates, more specifically the presence of a recombinant insert in the pUB110 plasmid, presumably also interfered with the ability of Canu, a well-established assembler, to provide a correct result (i.e. correct representation of free high-copy pUB110 plasmid carrying the wild-type protease gene). In addition to the evidence discussed in section 3.2, i.e. the issues with Unicycler assembly being attributed to the presence of a transgenic construct, this indicates that correct assembly of a GMM, even with long read data, can pose specific challenges, hence requiring a particularly careful approach including multiple additional *ad hoc* analyses instead of relying on the output of one particular assembler.

3. References

Berbers, B., Saltykova, A., Garcia-Graells, C., Philipp, P., Arella, F., Marchal, K., Winand, R., Vanneste, K., Roosens, N. H. C., & de Keersmaecker, S. C. J. (2020). Combining short and long read sequencing to characterize antimicrobial resistance genes on plasmids applied to an unauthorized genetically modified *Bacillus*. *Scientific Reports*, 10, Article 4310. https://doi.org/10.1038/s41598-020-61158-0.

Browne, P. D., Nielsen, T. K., Kot, W., Aggerholm, A., Gilbert, M. T. P., Puetz, L., Rasmussen, M., Zervas, A., & Hansen, L. H. (2020). GC bias affects genomic and metagenomic reconstructions, underrepresenting GC-poor organisms. *GigaScience*, 9, Article giaa008. https://doi.org/10.1093/gigascience/giaa008.

Chen, Z., Erickson, D. L., & Meng, J. (2020). Benchmarking hybrid assembly approaches for genomic analyses of bacterial pathogens using Illumina and Oxford Nanopore sequencing. *BMC Genomics*, 21, Article 631. https://doi.org/10.1186/s12864-020-07041-8.

Deckers, M., Deforce, D., Fraiture, M. A., & Roosens, N. H. C. (2020). Genetically modified micro-organisms for industrial food enzyme production: An overview. *Foods*, 9, Article 326. https://doi.org/10.3390/foods9030326.

de Maio, N., Shaw, L. P., Hubbard, A., George, S., Sanderson, N. D., Swann, J., Wick, R., Oun, M. A., Stubberfield, E., Hoosdally, S. J., Crook, D. W., Peto, T. E. A., Sheppard, A. E., Bailey, M. J., Read, D. S., Anjum, M. F., Sarah Walker, A., & Stoesser, N. (2019). Comparison of long-read sequencing technologies in the hybrid assembly of complex bacterial genomes. *Microbial Genomics*, 5, Article e000294. https://doi.org/10.1099/mgen.0.000294.

Fiedler, G., Kabisch, J., Brinks, E., Sprotte, S., Boehnlein, C., & Franz, C. M. A. P. (2020). Complete genome sequence of a Shiga toxin-producing *Escherichia coli* O26:H11 strain (sequence type 21) and two draft genome sequences of *Listeria monocytogenes* strains (clonal complex 1 [CC1] and CC59) isolated from fresh produce in Germany. *Microbiology Resource Announcements*, 9, Article e00973. https://doi.org/10.1128/mra.00973-20.

Gillis, A., & Mahillon, J. (2014). Phages preying on *Bacillus anthracis*, *Bacillus cereus*, and *Bacillus thuringiensis*: Past, present and future. *Viruses*, 6, 2623–2672. https://doi.org/10.3390/v6072623.

Gruss, A., & Ehrlich, S. D. (1988). Insertion of foreign DNA into plasmids from gram-positive bacteria induces formation of high-molecular-weight plasmid multimers. *Journal of Bacteriology*, 170, 1183–1190. https://doi.org/10.1128/jb.170.3.1183-1190.1988.

Harris, R., Dussault, F., Flint, A., Austin, J. W., & Weedmark, K. (2021). Complete genome sequence of *Clostridium botulinum* CJ0611A1, a type A(B) isolate associated with an international outbreak of botulism from commercial carrot juice. *Microbiology Resource Announcements*, 10, Article e01111. https://doi.org/10.1128/mra.01111-20.

Krishnakumar, R., Sinha, A., Bird, S. W., Jayamohan, H., Edwards, H. S., Schoeniger, J. S., Patel, K. D., Branda, S. S., & Bartsch, M. S. (2018). Systematic and stochastic influences on the performance of the MinION nanopore sequencer across a range of nucleotide bias. *Scientific Reports*, 8, Article 3159. https://doi.org/10.1038/s41598-018-21484-w.

Kurittu, P., Khakipoor, B., Aarnio, M., Nykäsenoja, S., Brouwer, M., Myllyniemi, A.-L., Vatunen, E., & Heikinheimo, A. (2021). Plasmid-borne and chromosomal ESBL/AmpC genes in *Escherichia coli* and *Klebsiella pneumoniae* in global food products. *Frontiers in Microbiology*, 12, Article 592291. https://doi.org/10.3389/fmicb.2021.592291.

Leenhouts, K. J., Kok, J., & Venema, G. (1990). Stability of integrated plasmids in the chromosome of *Lactococcus lactis*. *Applied and Environmental Microbiology*, 56, 2726–2735. https://doi.org/10.1128/aem.56.9.2726-2735.1990.

Viret, J. F., & Alonso, J. C. (1988). A DNA sequence outside the pUB110 minimal replicon is required for normal replication in *Bacillus subtilis*. *Nucleic Acids Research*, 16, 4389–4406. https://doi.org/10.1093/nar/16.10.4389.

Viret, J. F., Bravo, A., & Alonso, J. C. (1991). Recombination-dependent concatemeric plasmid replication. *Microbiological Reviews*, 55, 675–683. https://doi.org/10.1128/mmbr.55.4.675-683.1991.

Supplementary Figures

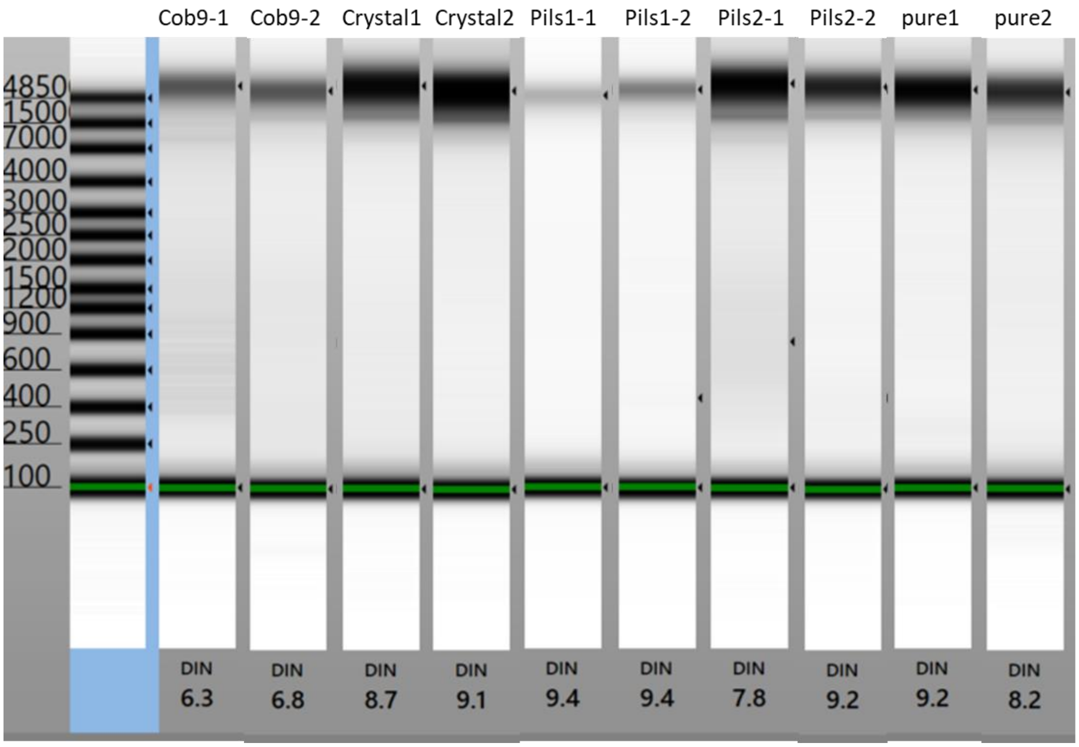

Figure S1. Visualization of DNA extracted from isolates of the protease-producing GM *B. velezensis* strain. The molecular-weight size marker is going from 100 to 48,500 bp. DIN is DNA Integrity Number.

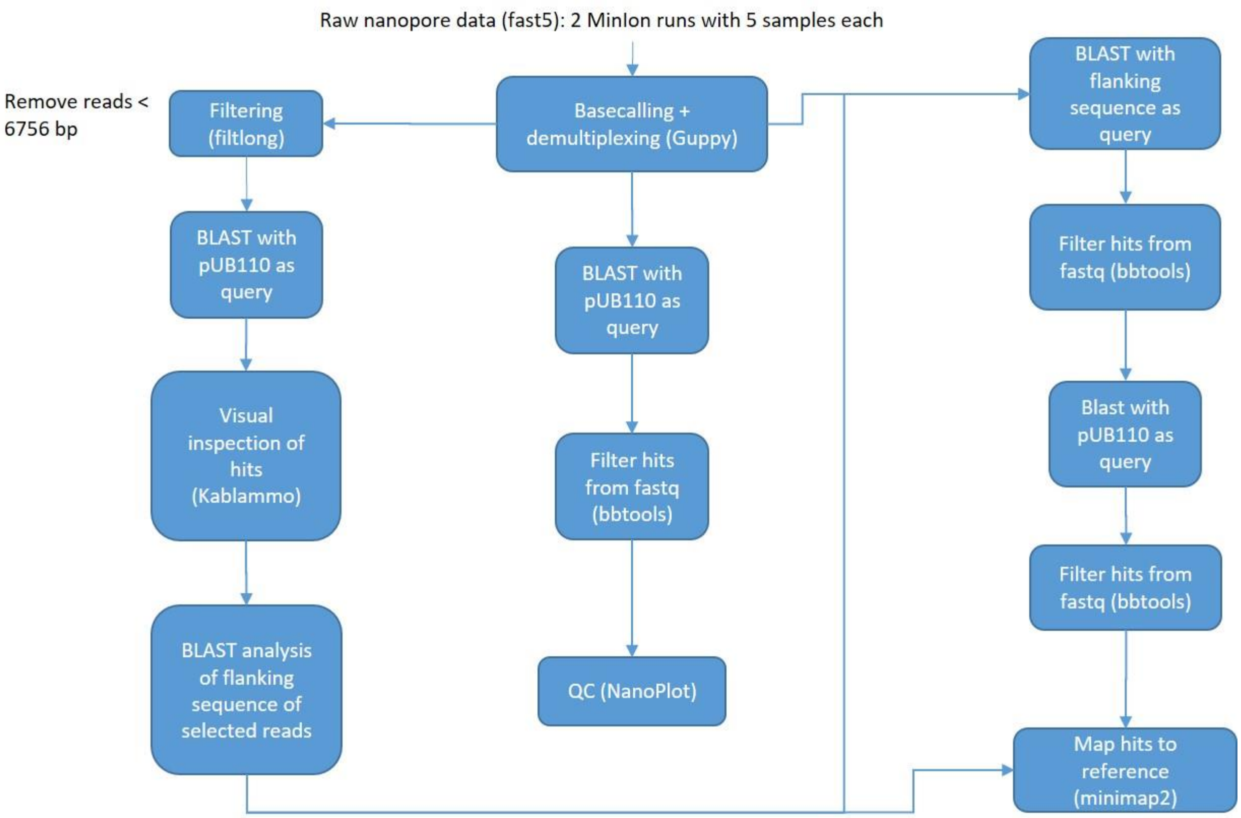

Figure S2. Workflow for analysis of raw long reads, as described in Material and methods section 2.5

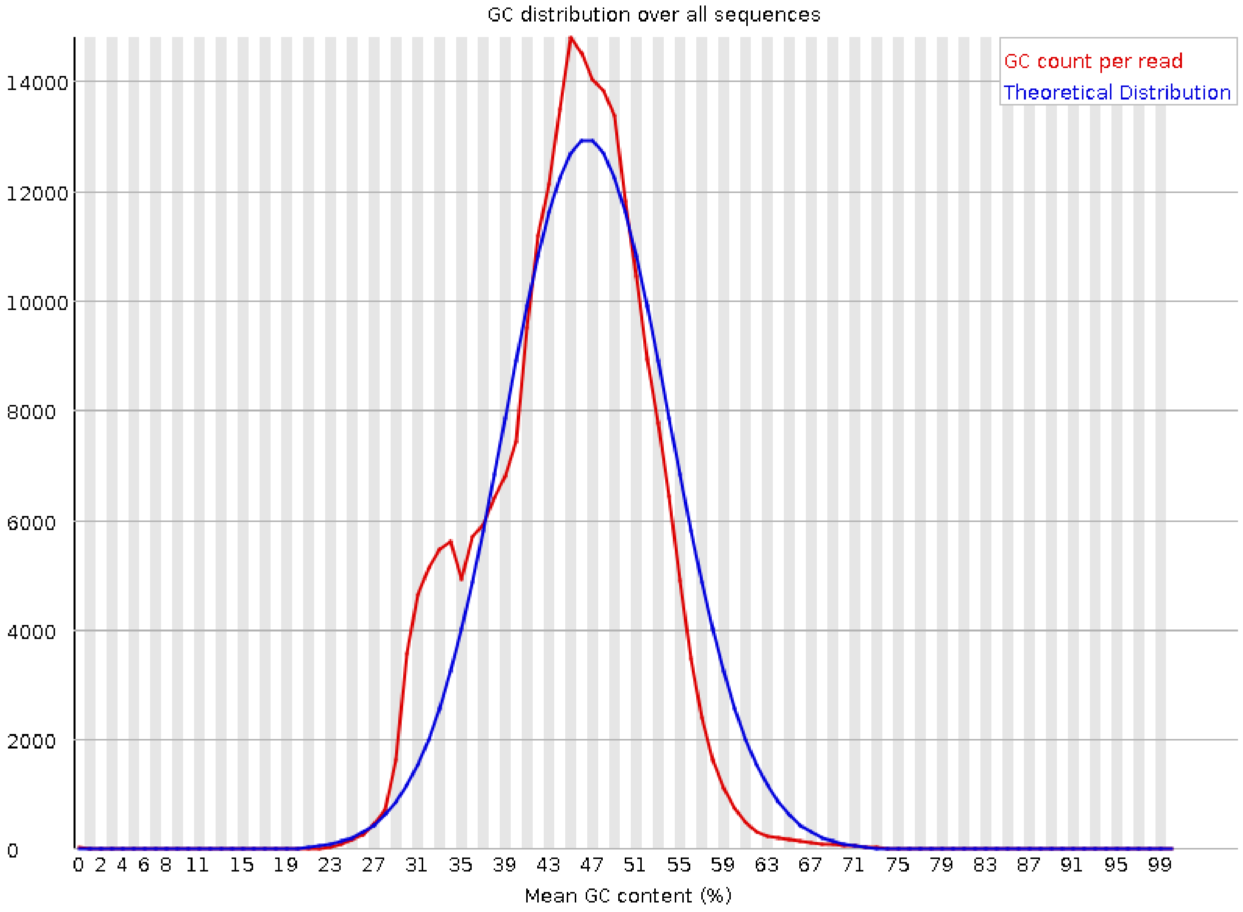

Figure S3. Per sequence GC content of raw forward short reads from isolate Cob9-1 (output of FastQC). A similar pattern was observed for the other isolates.

Assembly	Unicycler	Canu	hybridSPAdes
Cob9-1		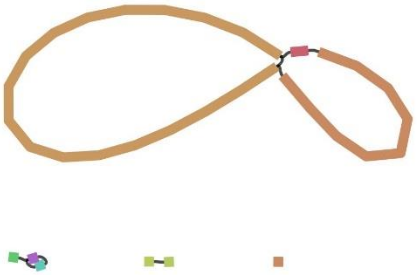	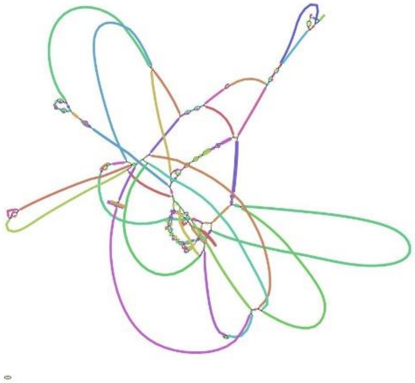
Cob9-2		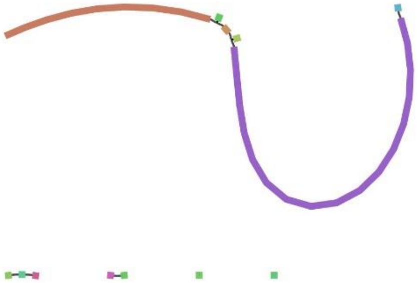	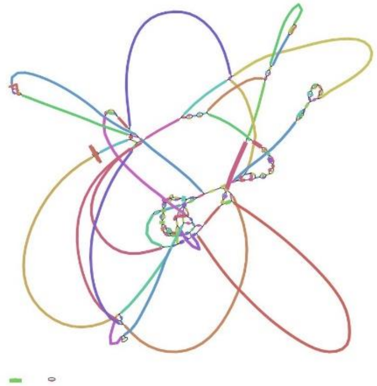

Crystal-1		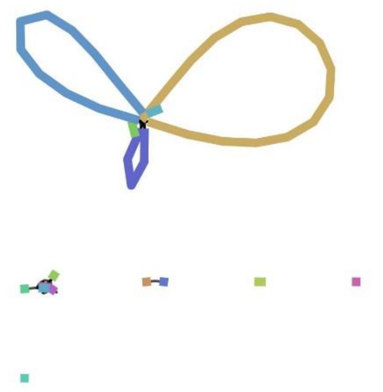	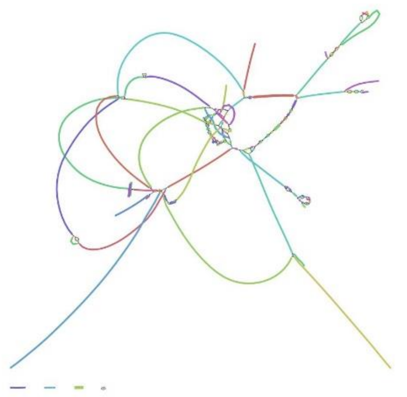
Crystal-2		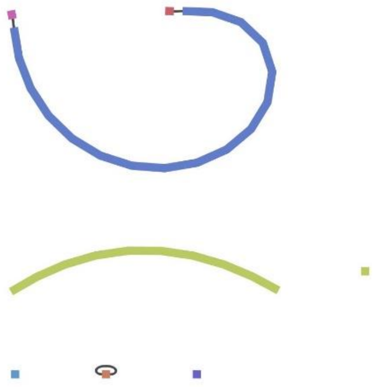	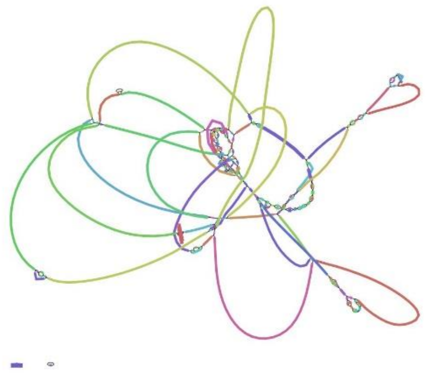

Pilsner1-1	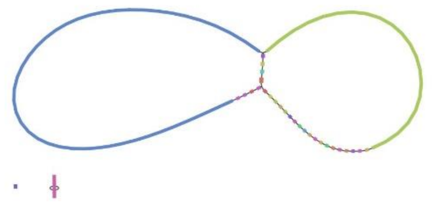	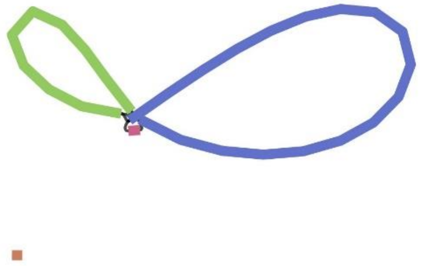	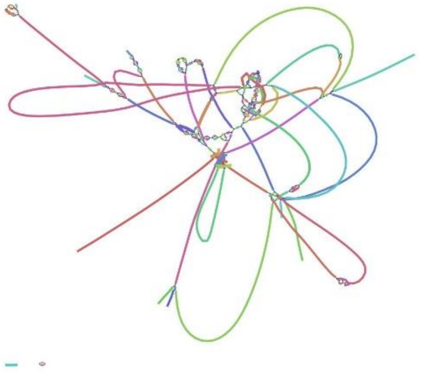
Pilsner1-2	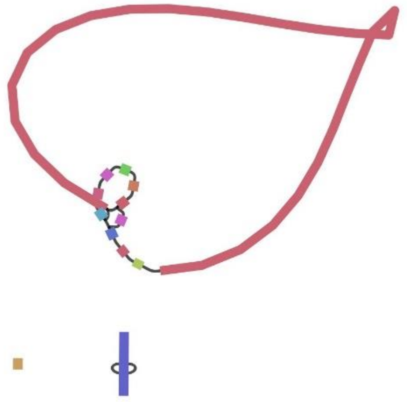	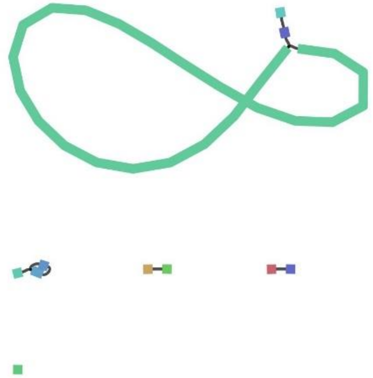	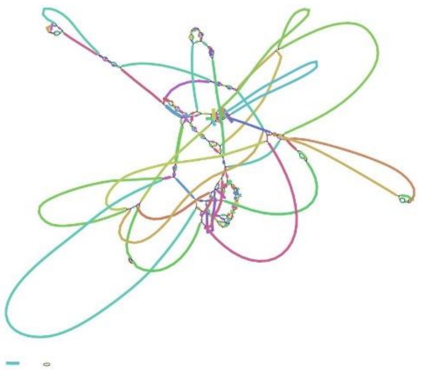

Pilsner2-1		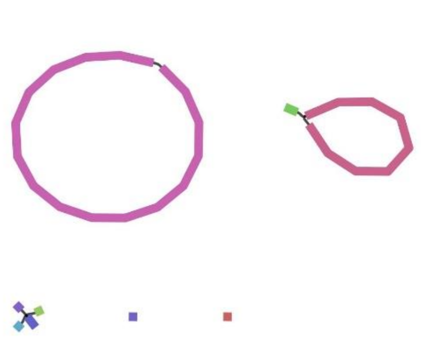	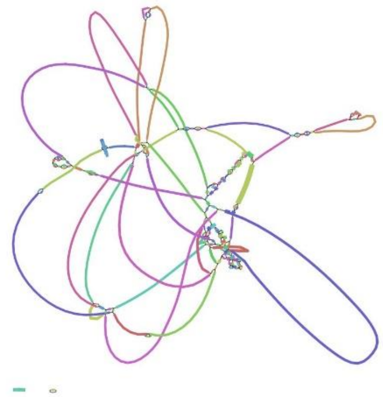
Pilsner2-2	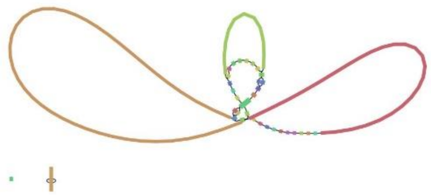	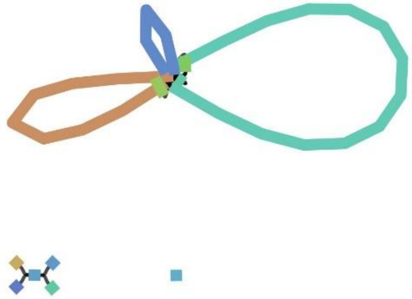	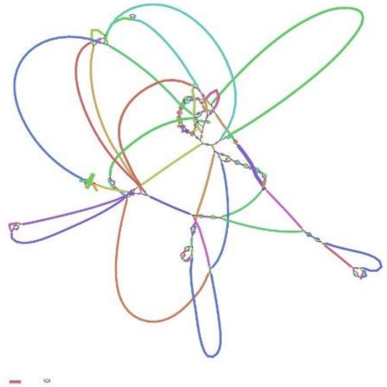

Pure-1		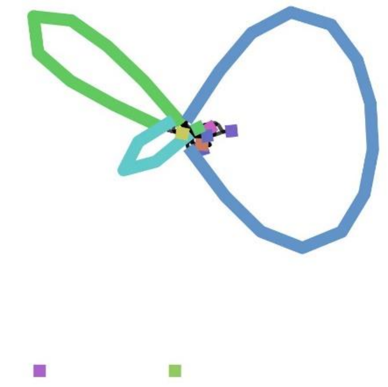	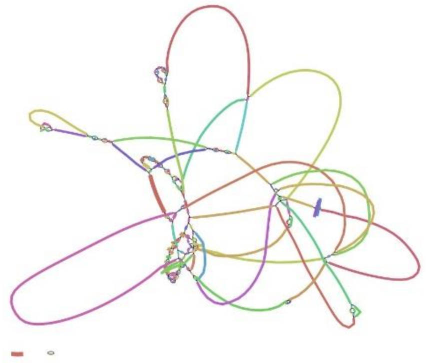
Pure-2		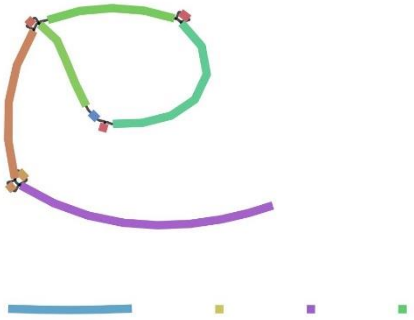	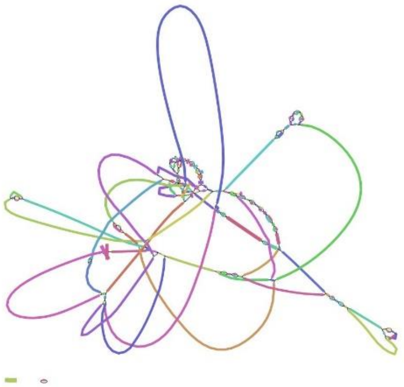

Figure S4. Assembly graphs, created with Bandage, for all successful assemblies

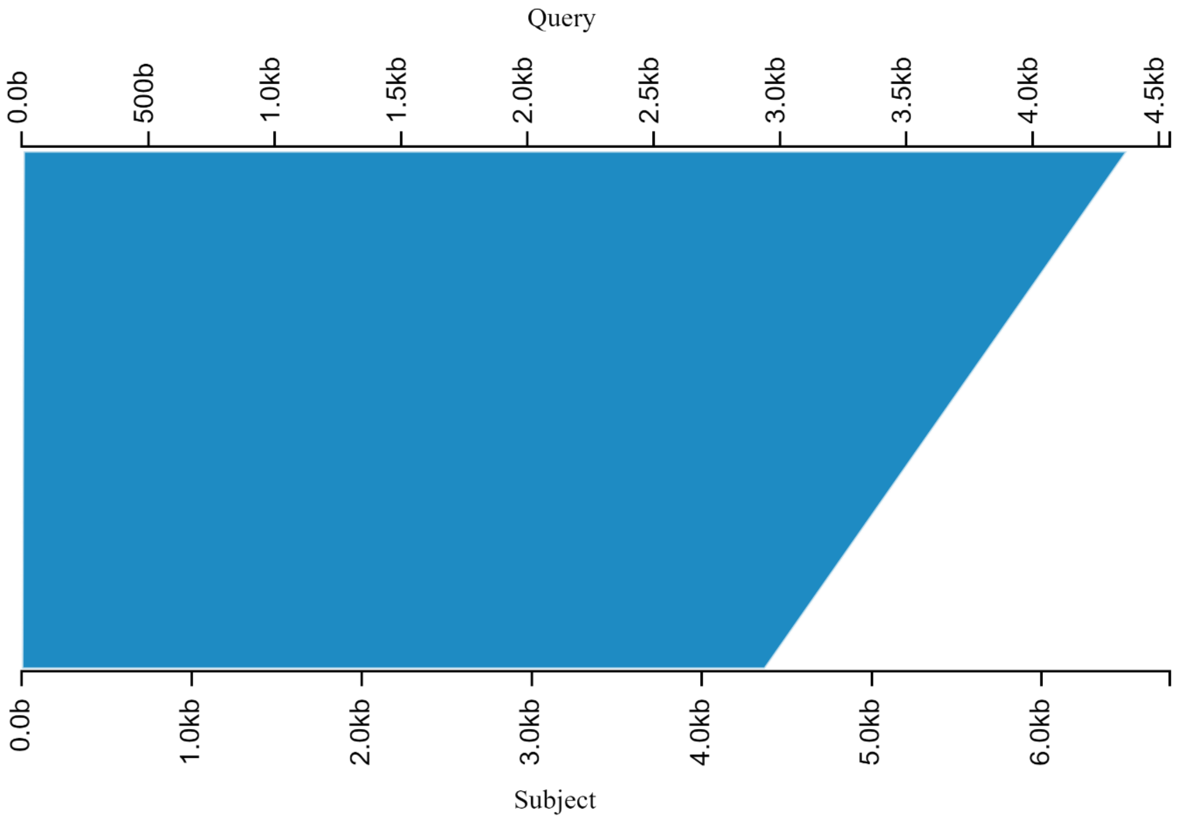

Figure S5. Alignment of the native pUB110 vector to the recombinant pUB110-derived plasmid from the GM isolates (created with Kablammo). The top ruler (query) represents the native plasmid, and the bottom ruler the recombinant plasmid. The blue zone indicates the aligned regions.

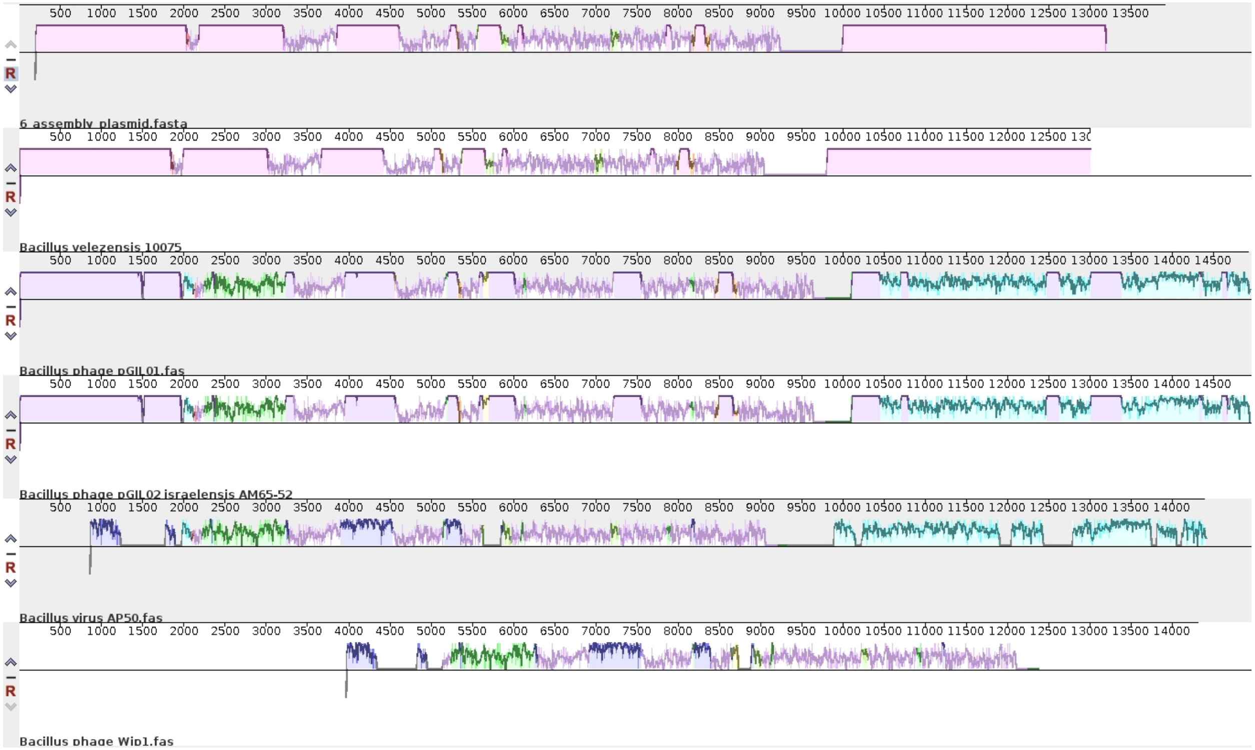

Figure S6. Multiple genome alignment of known and putative linear extrachromosomal prophages from different *Bacillus* strains (alignment conducted with ProgressiveMauve, visualization in Mauve alignment viewer). From top to bottom: linear ~14kb contig of isolate *B. velezensis* Pilsner1-2 unicycler assembly, component annotated as 'plasmid, unknown' from *B. velezensis* 10075 (Genbank: CP025940.1), *B. thuringiensis* phage pGIL01 (Genbank: CP013282), *B. thuringiensis* phage pGIL02 (Genbank: AJ536073), *B. anthracis* phage AP50 (Genbank: EU408779), *B. anthracis* phage Wip1 (Genbank: KF188458). Purple regions are conserved among all genomes. Other colored regions indicate sequence similarity that is not conserved among all genomes.

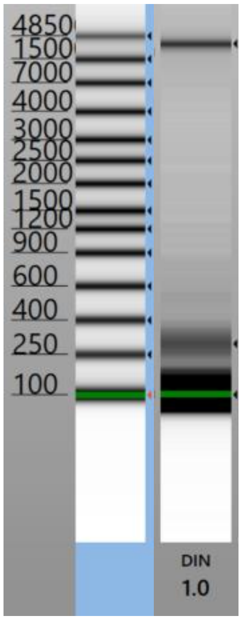

Figure S7. Intact plasmid DNA from GM bacterial isolate Cob9-1, visualized by capillary electrophoresis using the Tapestation 4200 device.

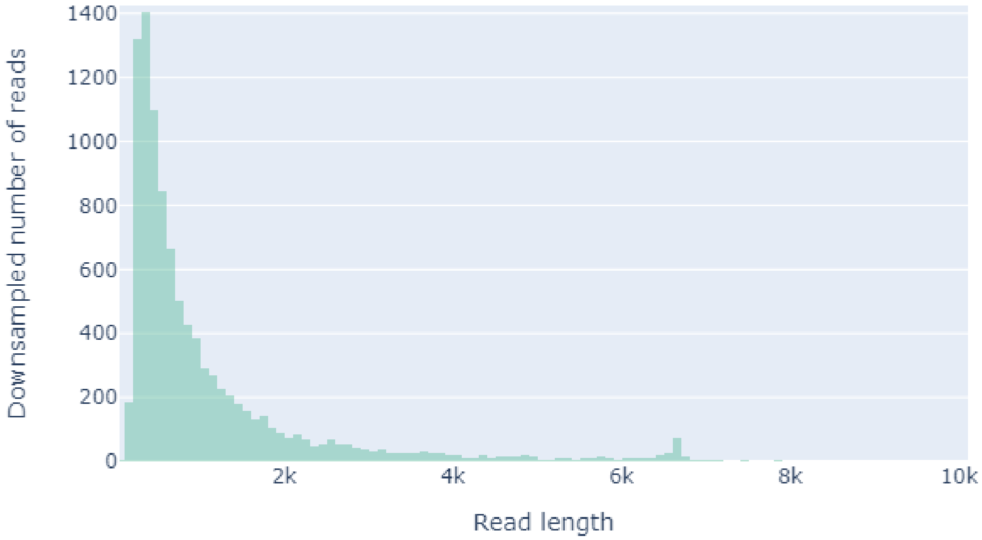

Figure S8. Read length profile of Cob9-1 raw long read data filtered to retain only reads matching pUB110 sequence (Dynamic read length histogram of NanoPlot, zoomed in to area of interest). The histogram reveals a distinct peak at 6.6–6.8 kbp, which supports the presence of the recombinant plasmid. For all the other isolates, a similar pattern was observed (results not shown).

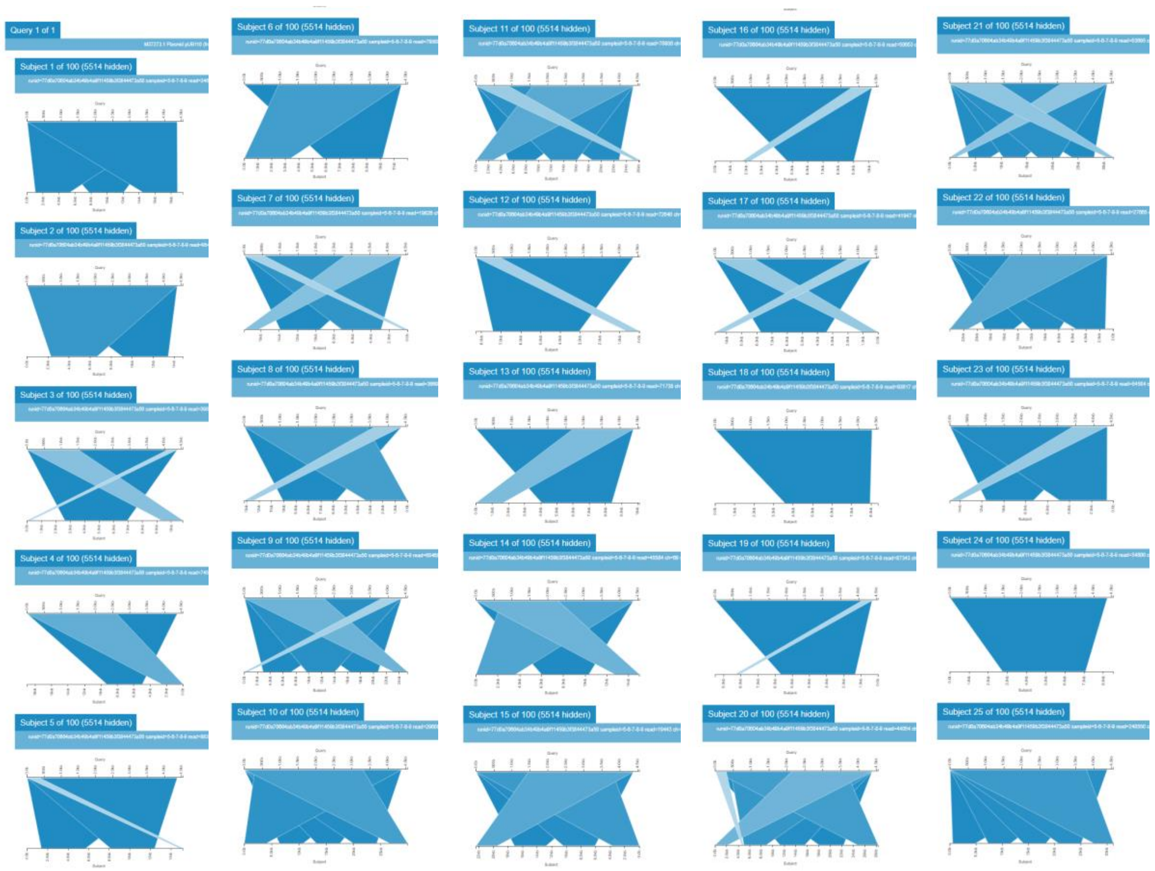

Figure S9. Top 25 hits of raw long reads of sample Pilsner1-2, BLAST analyzed using pUB110 as query, visualized with Kablammo. The ruler at the top of each diagram represents the query, i.e. the 4,545 bp sequence of the complete native pUB110 plasmid (Genbank: M19465.1). The bottom ruler represents the subject, i.e. the reads that matched with pUB110 sequence in the BLAST search. In almost all cases, the reads represent pUB110 concatemers. The regions in between the pUB1110-matching parts of the sequence represent the insert of the recombinant pUB110-derived plasmid described in this study.

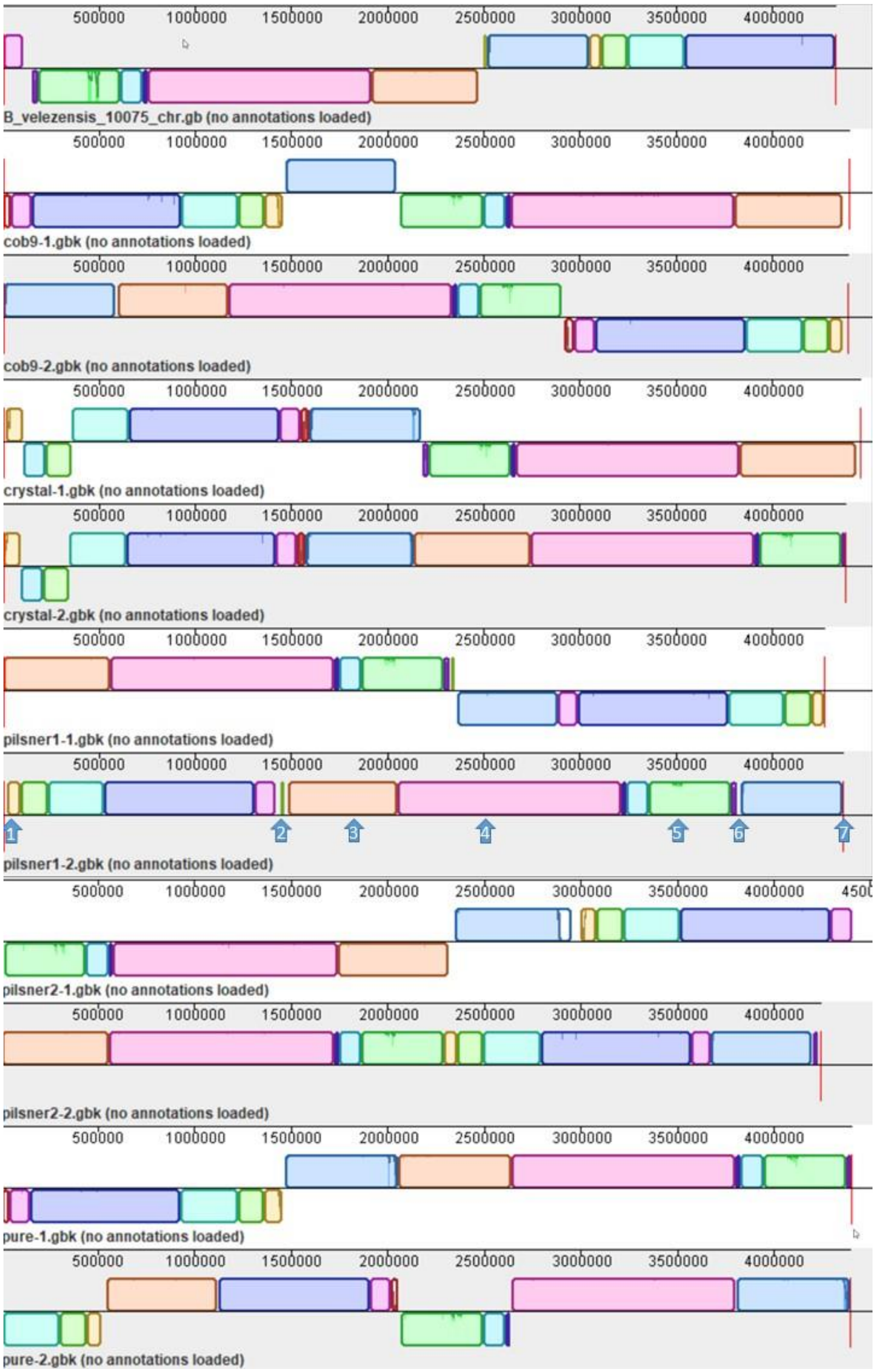

Figure S10. Multiple genome alignment, visualized with Mauve viewer, of the 10 GM isolates and reference strain *B. velezensis* 10075. The colored blocks are locally collinear blocks (LCBs), which represent regions of conserved continuous sequence similarity among the strains included in the alignment. Contig boundaries are marked with vertical red lines. Blue arrows on the track of sample Pilsner1-2 indicate approximate locations of the prophages detected on the largest chromosome contig with PHASTER analysis.

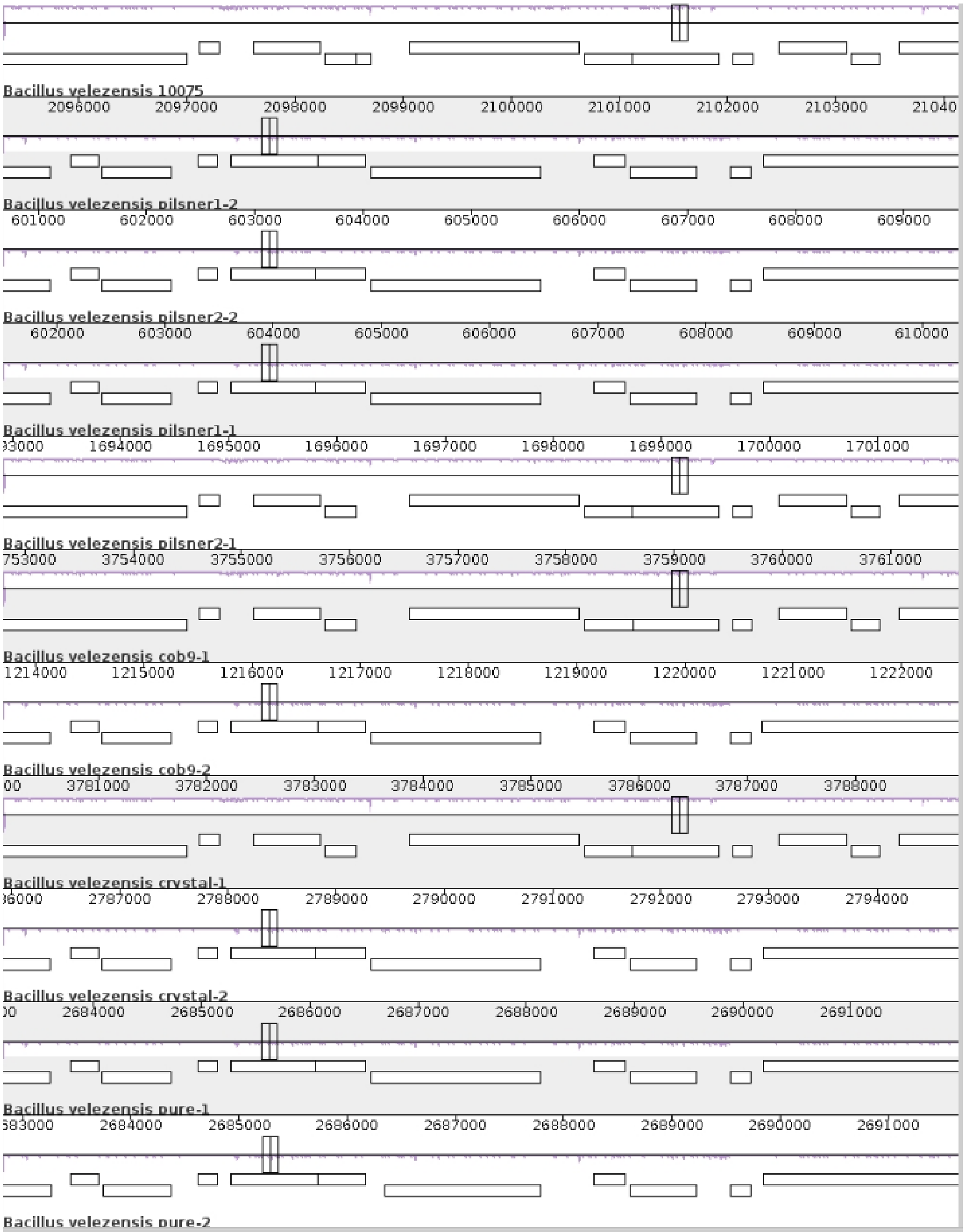

Figure S11. Whole genome comparison of 10 isolates and *B. velezensis* 10075, zoomed in on area around chromosomal protease encoding gene. The purple highlighting of the entire area indicates a high level of sequence conservation among all the genomes included in the alignment. Genome annotation is visualized under the sequence conservation track, and the mouse cursor (black vertical rectangle) highlights the position of the protease encoding gene.

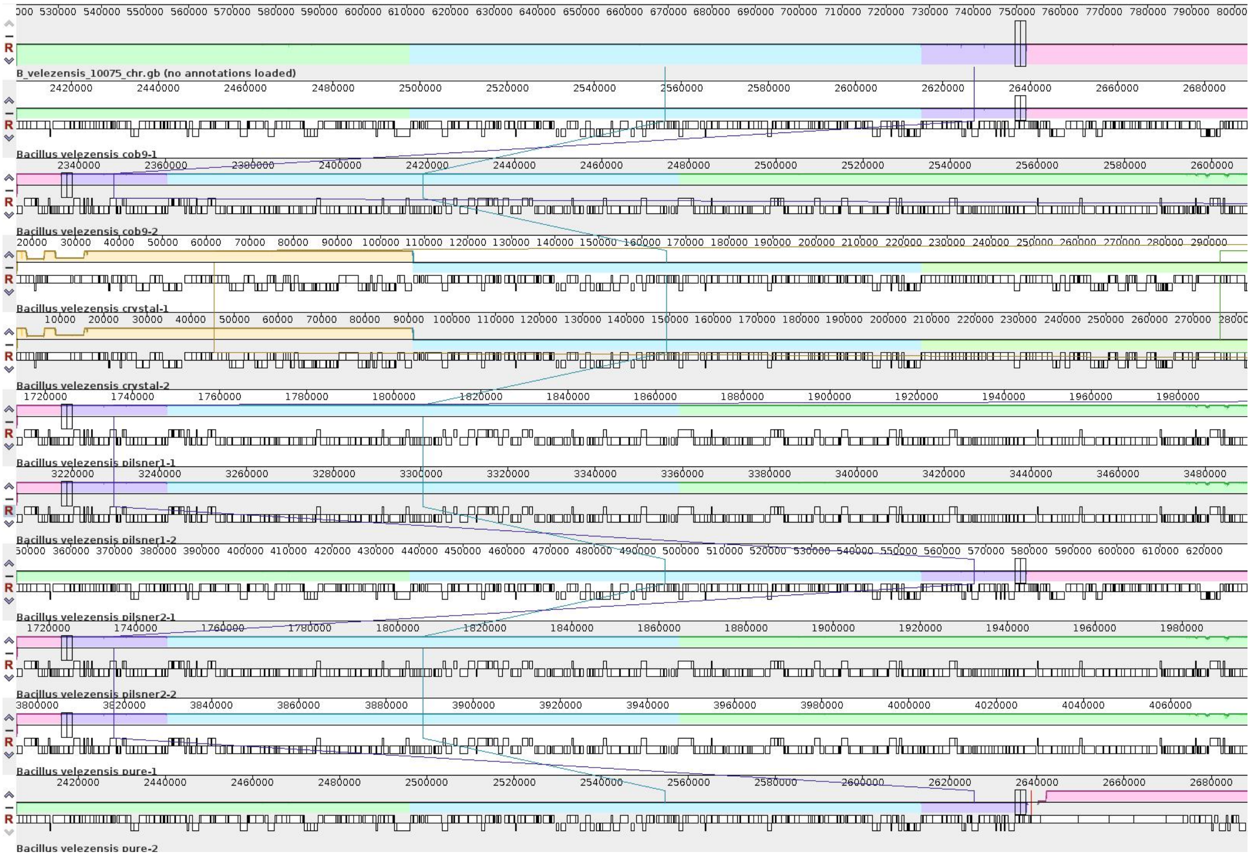

Figure S12. Mauve alignment view of *B. velezensis* 10075 (uppermost track) and 10 GM isolates, showing a number of locally colinear blocks (LCBs), which represent regions of continuous sequence conservation among the different genomes. The alignment view is centered on the light blue LCB. For most strains, including reference *B. velezensis* 10075, the light blue LCB is positioned between a purple and green LCB. For the Crystal strains however, the context is different for this light blue LCB, which is evident by its position between an orange and another green LCB.

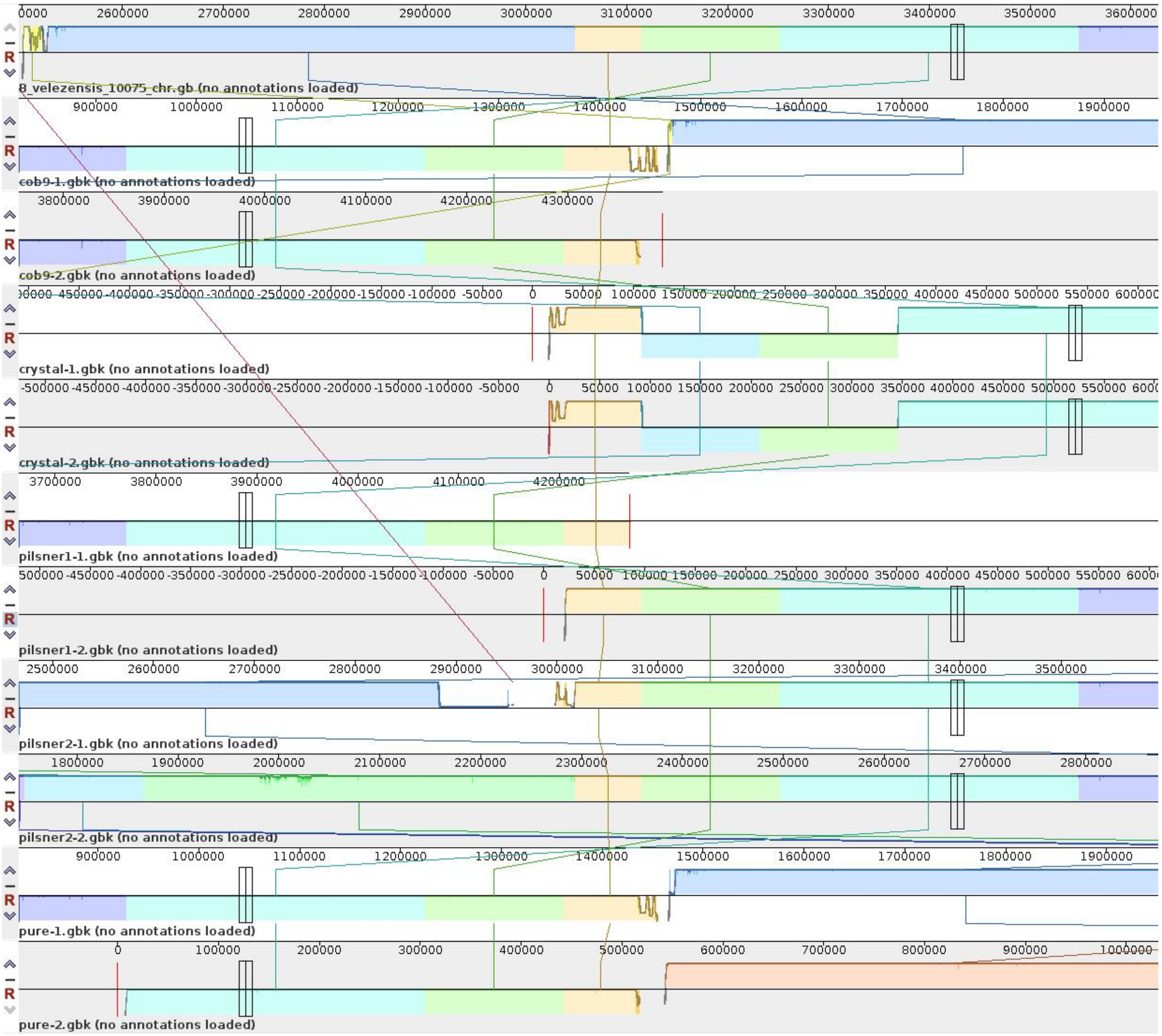

Figure S13. Mauve alignment view of the same multiple genome alignment as presented in Figure S 12, from another vantage point. In this figure, the view is centered on a light orange LCB. For isolates Crystal1 and Crystal-2 this is in essence the same view as in Figure S 12, where an orange LCB and a green LCB flank the light blue LCB of interest. However, in the other 8 GM isolates, the light blue LCB is not present, and the orange and green LCB are adjacent to each other.

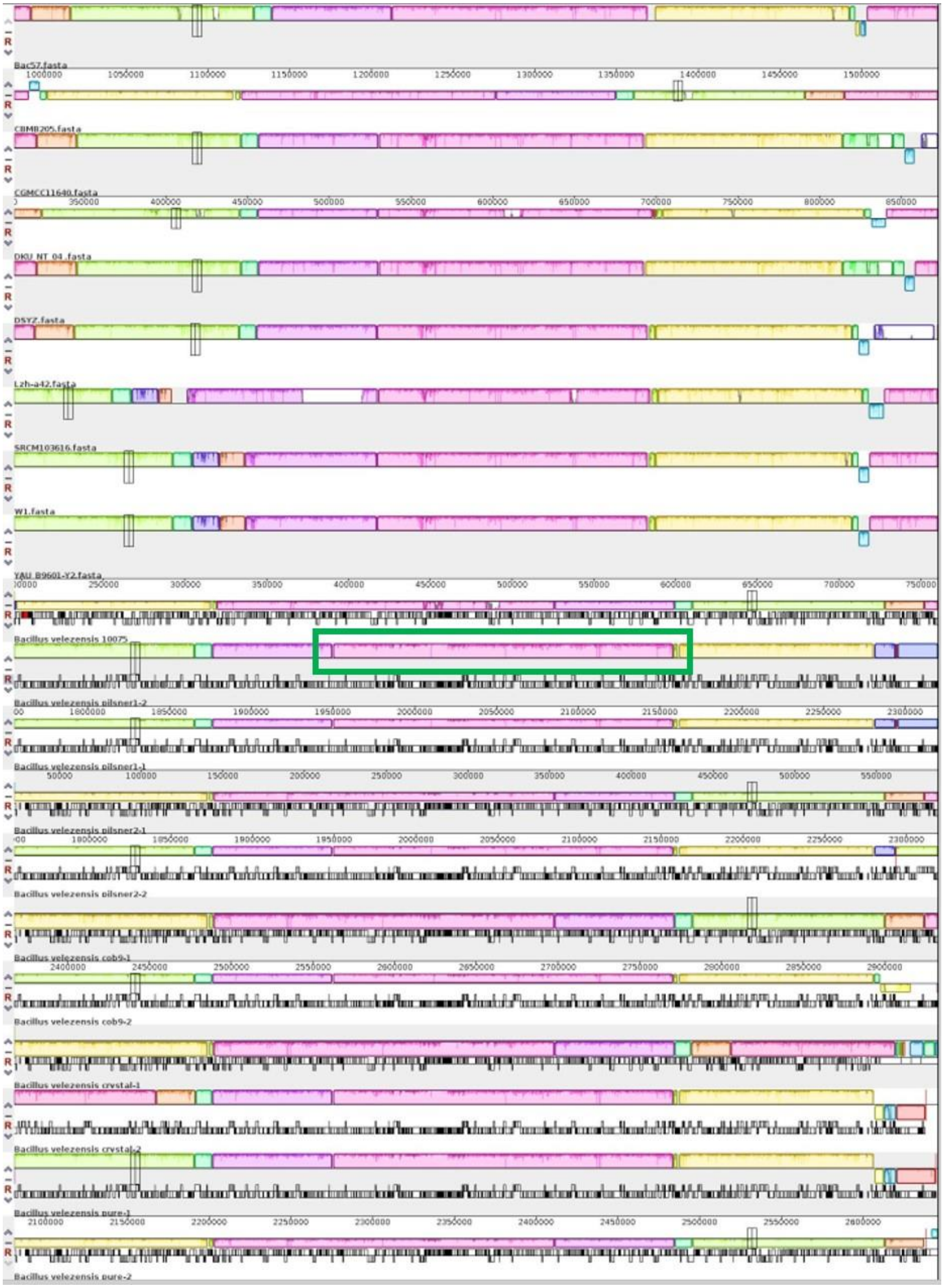
Bac57.fasta
CBMB205.fasta
CGMCC11640.fasta
DKU_NT_04.fasta
DSYZ.fasta
Lzh-a42.fasta
SRCM103616.fasta
W1.fasta
YAU_B9601-Y2.fasta
Bacillus velezensis 10075
Bacillus velezensis pilsner1-2
Bacillus velezensis pilsner1-1
Bacillus velezensis pilsner2-1
Bacillus velezensis pilsner2-2
Bacillus velezensis cob9-1
Bacillus velezensis cob9-2
Bacillus velezensis crystal-1
Bacillus velezensis crystal-2
Bacillus velezensis pure-1
Bacillus velezensis pure-2

Figure S14. Mauve viewer snapshot of multiple genome alignment of, from top to bottom: *B. velezensis* strains Bac57 (NZ_CP033054) , CBMB205 (NZ_CP011937), CGMCC11640 (NZ_CP026610), DKU_NT_04 (NZ_CP026533), DSYZ (NZ_CP030150), Lzh-a42 (NZ_CP025308), SRCM103616 (NZ_CP035410), W1 (NZ_CP028375),YAU B9601-2 (NZ_017061), 10075, and the GM isolates; Pilsner1-2 (designated as reference in the viewer), Pilsner1-1, Pilsner2-1, Pilsner2-2, Cob9-1, Cob9-2, Crystal-1, Crystal-2, Pure-1, Pure-2. According to the PHASTER analysis of Pilsner1-2, the pink LCB (framed in green on the track of Pilsner1-2) contains a putative prophage sequence (i.e. region 5 in Table S8), and the alignment shows that it is also present in the other GM isolates, as well as in *B. velezensis* 10075, although for the latter sequence similarity of the prophage region is markedly lower compared to among the isolates. However, the prophage is lacking from the other *B. velezensis* strains that were included in this alignment, which is apparent from the smaller size of the pink LCB in these strains, compared to the GM isolates and strain 10075.

Supplementary tables

Table S1. Metrics of Unicycler[1], hybridSPAdes, and Canu assemblies for the 10 GM isolates.

	Assembler	# contigs	N50 (bp)	GC%	Total length (bp)	Length plasmid[2] (bp)	Length putative prophage[2] (bp)
Cob9-1	Canu	9	2,944,648	46.08	4,564,503	/	/
	hybridSPAdes	173	494,018	45.98	4,267,374	/	/
Cob9-2	Canu	13	2,930,683	45.89	4,784,252	/	/
	hybridSPAdes	165	453,548	45.97	4,278,920	/	14,059
Crystal-1	Canu	15	1,581,208	45.87	4,860,151	/	14,040/14,048[3]
	hybridSPAdes	182	414,325	45.97	4,280,926	/	14,052
Crystal-2	Canu	8	2,812,617	46.03	4,559,075	/	14,046
	hybridSPAdes	171	522,603	45.97	4,281,016	/	14,060
Pilsner1-1	Canu	4	2,940,385	46.07	4,532,226	/	/
	hybridSPAdes	194	243,300	45.97	4,269,103	/	14,059
	Unicycler	31	2,884,838	46.05	4,365,515	6756	13,925
Pilsner1-2	Canu	11	4,466,927	45.88	4,706,574	/	/
	hybridSPAdes	195	361,438	45.97	4,285,375	/	14,054
	Unicycler	13	4,369,616	46.08	4,425,131	6756	13,899
Pilsner2-1	Canu	9	2,978,852	45.83	4,738,205	/	14,055
	hybridSPAdes	177	453,548	45.97	4,268,382	/	14,054
Pilsner2-2	Canu	12	1,478,348	45.96	4,854,307	/	14,053
	hybridSPAdes	177	519,746	45,97	4,267,101	/	14,054
	Unicycler	37	2,293,336	46,03	4,305,395	6756	13,922
Pure-1	Canu	12	2,352,274	46,17	4,678,891	/	/
	hybridSPAdes	171	453,548	45,97	4,280,704	/	14,059
Pure-2	Canu	15	594,720	46,03	4,657,343	/	/
	hybridSPAdes	174	519,745	45,97	4,281,562	/	14,057

[1]Absence of an Unicycler assembly for an isolate is due to failure of Unicycler to run to completion

[2]'/' signifies that the plasmid or prophage was not present in the assembly

[3]present twice in the assembly

Table S2. Overview of assemblies used as representative for each sample, and stats of pruned assemblies used in multiple genome alignment.

		Total assembly		Pruned assembly for multiple genome alignment		
**Isolate**	**Assembler**	**No contigs**	**Total length (bp)**	**No contigs**	**Total length (bp)**	**% of total assembly**
Cob9-1	Canu	9	4564503	2	4399633	96,4
Cob9-2	Canu	13	4784252	2	4391146	91,8
Crystal-1	Canu	15	4860151	3	4452477	91,6
Crystal-2	Canu	8	4559075	2	4375354	96,0
Pilsner1-1	Unicycler	31	4365515	2	4269246	97,8
Pilsner1-2	Unicycler	13	4425131	1	4369616	98,7
Pilsner2-1	Canu	9	4738205	2	4444201	93,8
Pilsner2-2	Unicycler	37	4305395	4	4242316	98,5
Pure-1	Canu	12	4678891	3	4397302	94,0
Pure-2	Canu	15	4657343	6	4392080	94,3

Table S3. Key metrics for Illumina and ONT raw data of this study

Sample	Short reads (raw)	Long reads (raw)		
	No reads	Read length N50	No reads	No bp
Cob9-1	461,014	1,927	2,422,322	2,669,456,564
Cob9-2	585,992	2,519	1,012,109	1,225,428,230
Crystal-1	678,097	5,120	1,472,000	1,739,007,378
Crystal-2	669,794	12,778	610,707	1,823,638,452
Pilsner1-1	461,536	12,809	594,424	1,469,480,606
Pilsner1-2	490,773	7,260	1,898,342	4,753,711,130
Pilsner2-1	564,948	2,342	1,199,536	1,481,107,575
Pilsner2-2	559,205	9,873	774,614	1,265,714,298
Pure-1	696,443	11,538	343,546	823,661,003
Pure-2	729,454	7,123	101,521	252,545,276

Table S4. *B. velezensis* strains included in the SNP phylogeny analysis

strain	run_accession	sample_accession	collection_date
BY-2	ERR4672761	SAMEA7370791	
Tu-100	ERR4674029	SAMEA7370792	
RASFF2019.3332	SRR10231466	SAMN12911415	01-01-19
GB03	SRR1034787	SAMN02413381	
WRB ZX-0001	SRR10397796	SAMN13160259	01-01-17
AL7	SRR10420793	SAMN13229277	01-10-08
CACC 316	SRR10916150	SAMN13885683	01-03-18
TNW2(2019)	SRR10969386	SAMN13927446	01-03-19
TNC2(2019)	SRR10969389	SAMN13927443	01-03-19
E68	SRR11344402	SAMN13937646	01-08-13
K1	SRR11470435	SAMN13916046	01-01-07
VRA_517_n	SRR11595324	SAMN13262483	01-08-19
VRA_336g_n	SRR11595325	SAMN13262482	01-08-19
VRA_517g_f	SRR11595330	SAMN13262495	01-08-19
VRA_336g_f	SRR11595331	SAMN13262494	01-08-19
S00166	SRR12416186	SAMN14908333	
SPL51	SRR12775027	SAMN14558907	01-01-18
BS89	SRR12962996	SAMN06007748	
V4	SRR3924094	SAMN05375008	20-10-11
CBMB205	SRR4187667	SAMN05192538	
P42	SRR4970268	SAMN05982126	01-06-14
A6	SRR4971602	SAMN05982127	01-08-14
RUPDJ	SRR5194627	SAMN05880571	
JW	SRR6505808	SAMN08389348	01-07-16
GF423	SRR7507268	SAMN09580239	

CE2	SRR7965938	SAMN10178429	01-01-16
OSY-GA1	SRR7990333	SAMN09856379	01-12-17
SW5	SRR8205397	SAMN10439687	05-08-16
DSYZ	SRR8252721	SAMN09354865	01-01-13
MG33	SRR8316559	SAMN08384465	01-01-17
MG43	SRR8316560	SAMN08384466	01-01-17
FH17	SRR8443425	SAMN10375213	01-01-17
TH16	SRR8443433	SAMN10375212	01-01-17
ZeaDK315Endobac16	SRR8483183	SAMN10813014	14-07-16
UTB96	SRR8769565	SAMN11037194	01-10-10
PG12	SRR8935607	SAMN08107518	01-06-11
PG12	SRR8935608	SAMN08107518	01-06-11
LP16S	SRR9023184	SAMN11373588	22-06-17
PJRB2	SRR9317475	SAMN11964574	18-08-09
UCMB5044	SRR9701317	SAMN12015793	30-09-90
UCMB5007	SRR9701319	SAMN12015780	30-06-89
At1	SRR9701323	SAMN12058370	20-05-00
F85	SRR9925238	SAMN12530383	01-01-16

Table S5. Metrics of CFSAN pipeline run on GM isolates under study, together with all *B. velezensis* strains for which paired-end Illumina data was publicly available (January '21), with the Unicycler assembly of isolate Pilsner1-2 as reference. The CFSAN pipeline filters the initially retrieved (unfiltered) set of SNPs to retain a highly reliable filtered subset of SNPs.

Sample	Total reads	Mapping rate (%)	Avg. pileup depth	Nb. of SNPs (unfiltered)	Nb. of SNPs (filtered)
A6	7,107,698	87.59	158.75	28,237	1
AL7	8,066,412	94.73	210.06	53,460	0
At1	34,562,286	91.62	596.18	51,286	0
BS89	1,052,328	91.74	28.81	48,339	0
BY-2	11,121,252	86.52	188.56	67,078	0
CACC_316	9,931,314	93.31	260.00	28,687	2
CBMB205	6,358,784	89.54	152.80	52,578	0
CE2	2,427,774	89.39	44.75	50,389	0
DSYZ	6,331,672	85.52	234.93	55,148	0
E68	49,571,106	83.06	552.66	51,419	1
F85	9,650,610	85.04	184.43	67,035	0
FH17	17,594,274	91.85	471.83	5,233	4
GB03	27,492,038	92.42	459.29	53,767	0
GF423	29,008,528	71.00	365.55	55,521	3
JW	8,646,154	84.31	163.66	27,570	2
K1	5,383,542	90.41	135.31	54,734	0
LP16S	19,028,982	93.61	536.38	26,050	3
MG33	12,851,474	86.69	264.62	55,049	1
MG43	10,979,128	91.51	238.73	53,169	0
OSY-GA1	3,634,406	89.74	70.12	63,885	0
P42	4,882,652	84.02	95.48	50,785	2

PG12	13,339,324	52.59	82.17	66,991	0
PJRB2	29,702,782	92.88	626.87	67,199	0
RASFF2019.3332	1,429,274	99.03	50.60	16	1
RUPDJ	7,784,450	89.09	190.93	51,916	0
S00166	11,256,052	87.86	211.85	66,904	0
SPL51	14,074,494	88.98	342.52	8,051	3
SW5	9,176,780	88.84	153.18	53,603	0
TH16	17,095,330	92.70	422.40	53,348	0
TNC22019	3,877,760	91.29	60.05	22,744	1
TNW22019	3,918,210	84.86	49.44	51,357	0
Tu-100	8,994,162	88.61	160.32	66,930	0
UCMB5007	32,405,476	90.62	546.39	53,670	1
UCMB5044	28,428,202	86.63	426.38	53,543	1
UTB96	1,907,244	86.52	39.01	63,875	2
V4	3,434,392	88.84	146.57	52,790	0
VRA_336g_f	1,309,100	88.24	25.16	62,980	0
VRA_336g_n	8,380,456	90.15	99.19	65,549	0
VRA_517_n	5,128,270	88.65	60.72	65,063	0
VRA_517g_f	364,452	77.04	7.18	55,704	0
WRB_ZX-0001	2,361,282	79.10	50.49	52,441	0
ZeaDK315Endobac16	14,381,978	87.94	353.88	52,886	0
cob9-1	922,028	98.64	32.93	19	1
cob9-2	1,171,984	98.84	37.14	19	1
crystal-1	1,356,194	99.03	41.37	16	1

crystal-2	1,339,588	99.03	40.19	16	1
pilsner1-1	923,072	99.14	34.22	2	0
pilsner1-2	981,546	99.01	36.01	1	0
pilsner2-1	1,129,896	98.89	39.38	19	1
pilsner2-2	1,118,410	99.01	34.88	18	1
pure-1	1,392,886	98.85	45.91	15	1
pure-2	1,458,908	98.94	50.81	21	1

Table S6. Metrics of CFSAN pipeline of selected sample set used for final SNP phylogeny. The CFSAN pipeline filters the initially retrieved (unfiltered) set of SNPs to retain a highly reliable filtered subset of SNPs.

Sample	Total reads	Mapping rate (%)	Avg. pileup depth	Nb. of SNPs (unfiltered)	Nb. of SNPs (filtered)
FH17	17,594,274	91.85	471.83	5,019	165
SPL51	14,074,494	88.98	342.52	7,670	219
cob9-1	922,028	98.64	32.93	19	14
cob9-2	1,171,984	98.84	37.14	19	15
crystal-1	1,356,194	99.03	41.37	16	14
crystal-2	1,339,588	99.03	40.19	16	14
pilsner1-1	923,072	99.14	34.22	2	2
pilsner1-2	981,546	99.01	36.01	1	1
pilsner2-1	1,129,896	98.89	39.38	19	16
pilsner2-2	1,118,410	99.01	34.88	18	15
pure-1	1,392,886	98.85	45.91	15	15
pure-2	1,458,908	98.94	50.81	21	16

Table S7. SNP distance matrix with pairwise distances for the 10 GM isolates

	Cob9-1	Cob9-2	Crystal-1	Crystal-2	Pilsner1-1	Pilsner1-2	Pilsener2-1	Pilsner2-2	Pure-1	Pure-2
Cob9-1	0	10	14	14	17	16	16	16	12	18
Cob9-2	10	0	16	16	19	18	18	18	14	20
Crystal-1	14	16	0	0	17	16	16	16	12	18
Crystal-2	14	16	0	0	17	16	16	16	12	18
Pilsner1-1	17	19	17	17	0	1	17	17	15	21
Pilsner1-2	16	18	16	16	1	0	16	16	14	20
Pilsner2-1	16	18	16	16	17	16	0	0	14	20
Pilsner2-2	16	18	16	16	17	16	0	0	14	20
Pure-1	12	14	12	12	15	14	14	14	0	16
Pure-2	18	20	18	18	21	20	20	20	16	0

Table S8. Summary of prophage analysis of complete Unicycler assembly of sample Pilsner1-2 with PHASTER. Completeness of prophage region is scored by PHASTER, e.g. based on similarity to known prophage genes, and evaluated as 'intact', 'questionable', or 'incomplete', depending on the weight of evidence supporting the presence of a prophage. The most common phage name is the phage in database for which the highest number of matching genes was found. In the column labeled 'region position', region 1 is the largest contig of the pilsner1-2 assembly chromosome scaffold, while region 2 is a smaller contig of the chromosomal scaffold. Region 3 is the separate linear contig from the Unicycler assembly, which is presumed to be a plasmidic prophage. According to PHASTER, it is most similar to GIL16c, a known *Bacillus* tectivirus.

Region	Region length (kb)	Completeness	Region position	Total protein #	Phage hit protein #	Att site	Most common phage name (hit genes count)	Most common phage %	GC %
1	33.8	questionable	1,length=4369616,depth=1.00x:433-34328	39	31	no	PHAGE_Bacill_PfEFR_4_NC_048641(6)	15.38%	47.80%
2	72	intact	1,length=4369616,depth=1.00x:1421753-1493764	98	72	yes	PHAGE_Paenib_Tripp_NC_028930(15)	15.3%	47.75%
3	33.1	intact	1,length=4369616,depth=1.00x:1834372-1867549	45	32	no	PHAGE_Brevib_Jimmer2_NC_041976(8)	17.77%	46.62%
4	136.2	questionable	1,length=4369616,depth=1.00x:2427635-2563838	191	122	yes	PHAGE_Bacill_SPbeta_NC_001884(69)	36.12%	35.91%
5	50.5	intact	1,length=4369616,depth=1.00x:3475265-3525796	80	54	yes	PHAGE_Aeriba_AP45_NC_048651(21)	26.25%	43.17%
6	75	intact	1,length=4369616,depth=1.00x:3780071-3855155	105	74	yes	PHAGE_Bacill_Mgbh1_NC_041879(15)	14.28%	47.43%
7	15.7	incomplete	1,length=4369616,depth=1.00x:4353137-4368903	9	6	yes	PHAGE_Paenib_Tripp_NC_028930(3)	33.33%	43.45%
8	13.9	incomplete	2,length=14349,depth=1.65x:167-14161	24	13	no	PHAGE_Bacill_SIOphi_NC_042133(2)	8.33%	47.70%
9	13.1	incomplete	3,length=13899,depth=2.42x:146-13319	24	18	no	PHAGE_Bacill_GIL16c_NC_006945(12)	50%	43.71%
